# Evolution of documents related to performance in boccia: a paralympic sport bibliometric analysis

**DOI:** 10.3389/fspor.2025.1560803

**Published:** 2025-06-17

**Authors:** Cátia C. Ferreira, Víctor Hernández-Beltrán, José M. Gamonales, Mário C. Espada, Jesús Muñoz-Jiménez

**Affiliations:** ^1^ Instituto Politécnico de Setúbal, Escola Superior de Educação, Setúbal, Portugal; ^2^ Optimization of Training and Sports Performance Research Group, Faculty of Sport Science, University of Extremadura, Cáceres, Spain; ^3^ Sport Physical Activity and Health Research & INnovation CenTer (SPRINT), Rio Maior, Portugal; ^4^ Faculty of Education and Psychology, University of Extremadura, Badajoz, Spain; ^5^ Comprehensive Health Research Centre (CHRC), University of Évora, Évora, Portugal; ^6^ Life Quality Research Centre (CIEQV-Setúbal), Setúbal, Portugal; ^7^ CIPER, Faculdade de Motricidade Humana, Universidade de Lisboa, Lisboa, Portugal

**Keywords:** paralympic, publication analysis, adapted sport, research, trend

## Abstract

**Background:**

The purpose of this study was to develop a bibliometric review of literature related to Boccia, aiming to analyse relevant information related to the adapted sport, and determine the existing relationships/networks between the different authors and countries, as well as methodological procedures of publications.

**Methods:**

The Web of Science database was used, considering the keywords “Boccia” OR “Bocha” AND “Sport”, applying the filter “topic” to identify documents containing this term in the title, abstract, or keywords. A total of 89 studies were found and 38 selected based on the criteria, published until November 2024.

**Results:**

From 2012 forward, the number of studies increased by 81.87% from 2017 to 2024, with a notable rise between 2017 and 2020. Two documents had 20 or more citations**,** and a total of 119 authors were associated with 38 selected documents, an average of 3.6 authors per document. Portugal and Spain present the highest number of documents (*n* = 9), but Spain has more than twice thecitations compared to Portugal (58 vs.17). From a total of 29 resources, *Springer Nature* had the highest number of studies indexed in its platforms (*n* = 7). A total of 92 keywords were identified, and the terms with the highest occurrence were “boccia” (*n* = 17), “rehabilitation” (*n* = 4), and “disability” (*n* = 4). Brazil and Indonesia were the countries with the most recent scientific production, and 90.6% of the studies were written in English and 9.4% in Spanish.

**Conclusions:**

The findings emphasize the limited research output in Boccia, particularly regarding performance analysis, coaching methodologies, and athlete development. Future studies should explore training strategies, physiological demands, and the impact of Boccia on athleteś development and social inclusion.

## 1 Introduction

In recent times, there has been a solid increase in the number of sports available for people with disabilities, at a recreational and competitive level, creating more opportunities for these athletes to participate in sports (1, 2). Adapted sports foster physical and emotional health, helping individuals with disabilities to focus on their potential (3, 4) and promoting numerous benefits to its practitioners, such as confidence and self-efficacy (1) and higher levels of quality and satisfaction with life compared with individuals not involved in sports (3, 5, 6).

Boccia is a sport that is inclusive of all genders, ages, and abilities (7). In sports such as Boccia, individuals compete intending to achieve success. This adapted sport aims to improve the quality of life of its participants and facilitate their integration into society (8). Beyond its physical benefits, Boccia emphasizes strategy, teamwork, and social interaction, which fosters emotional regulation, empathy, and interpersonal skills, contributing to the development of emotional intelligence (9).

This parasport was practiced for many years as a leisure activity, only introduced at the Stoke Mandeville and New York 1984 Paralympic Games as a competitive sport (10). Nowadays, people with cerebral palsy (CP) can compete in individual classes BC1, BC2 or BC3 (11), the BC4 individual class is associated to “athletes who are diagnosed with an impairment of non-neurological origin not affecting the central nervous system and who do not present tonal change or spasticity as their primary impairment” (12). This precision adapted sport is played on a court with two sides, each side has six balls (red or blue), and players must throw these balls, using a method determined by their classification, out into the playing zone and as close as possible to the jack (the white ball on a playing hard surface court measuring 12.5 × 6 m (13). Boccia can also be played in pairs or teams, and BC3 players receive assistance during the throw or resort to a ramp. By participating in activities such as Boccia, individuals with physical disabilities become active participants in community life (9). The outcome of the study conducted by Siavoshy and Bolurian (14), focusing on the effect of practicing Boccia on the social development of children with cerebral palsy and intellectual disability, showed that the intervention could significantly improve self-help in general, self-help eating, verbal communication, socialization, and locomotion. Unfortunately, the literature draws attention to the lack of sufficient research on the important components of training para-athletes or adapted sports (15), which in our perspective is related to the lower number of athletes compared to sports and less media impact (for example on television), situations that seem to us to be improving in recent years in adapted sports, in general.

In addition, contact with the condition of disability and its clinical and social particularities has always created some barriers in society, which in our view have been broken down over the years, and we are currently seeing athletes with disability condition training and competing with athletes without a disability condition, which poses challenges in the training process and competitive moments. Boccia requires sustained concentration, and it is well established that regular mental stimulation improves the quality of life and independence (16). When individuals are playing Boccia, they have the opportunity to improve functionality and to interact with people with similar life problems. This adapted sport serves as a means of engagement for individuals with all types of disabilities, and based on studies, improves functional health and quality of life, helping in the inclusion/integration process in society (17).

Boccia has excellent effects on motor skills, and Williamson et al. (18) argued that the most important factor during the training of athletes with a disability is the coach's knowledge of aspects related to rehabilitation, skill level, awareness, and knowing how to provide the athlete with constant safety. Very little research has reported scientific evidence related to Boccia athletes. There is a great space and need for further research, particularly regarding “Coaching Science”, to improve training conditions and optimize sports performance (19). Therefore, the main objective of this study was to develop a bibliometric review of the literature related to Boccia, aiming to analyse information related to the adapted sport (publications, citations, journals, keywords), and determine the existing relationships/network between the different authors and countries that have participated in publications related to Boccia.

## 2 Materials and methods

### 2.1 Study design

The main objective of the present work was to develop a bibliometric review of the existing literature, which is framed within the Theoretical Studies (20). In addition, due to the evaluation of existing information, it is classified within the studies with retrospective methodology (21). This method allows obtaining more information related to the research topic, as well as understanding the state of the art (22). In the same way, it allows collecting documents from the same database, as well as filtering and refining the search simply (23). Therefore, the following phases were followed for the elaboration of the present study: (1) selection of the topic, (2) selection of the research method, (3) collection of information, (4) analysis of the information, (5) visualization, and (6) interpretation.

### 2.2 Search strategy

To identify the documents, the following search phrase was used: “Boccia” OR “Bocha” AND “Sport” using the filter “topic” to identify those documents that contained this term in the title, abstract, or keywords. In addition, in order to select the largest number of documents related to the study topic, the documents had to be related to Boccia as the main topic of the study, that is, the research question had to be focused on the study of an area of this modality.

**Figure 1:**
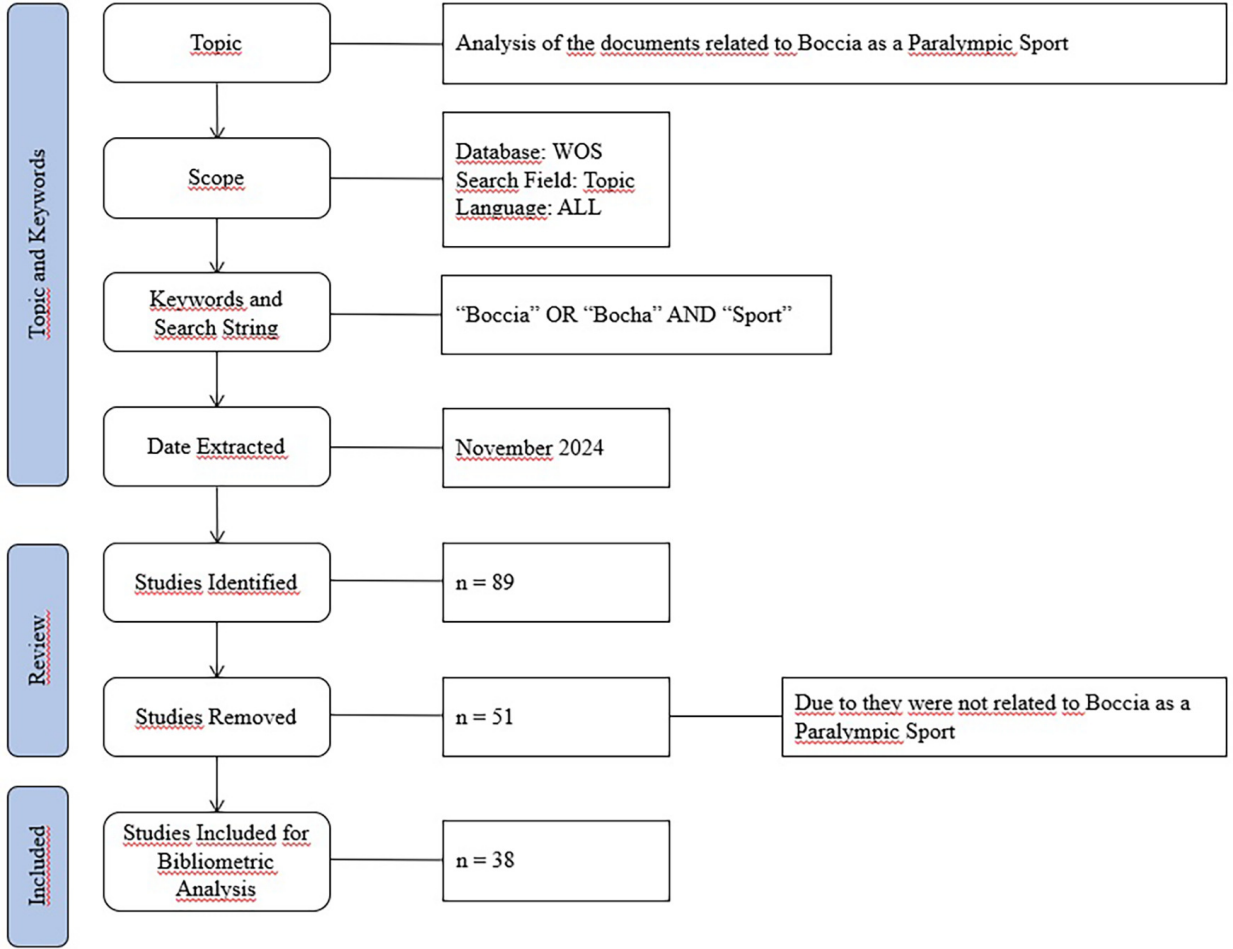
Flow chart of the document search.

To this end, the search was carried out with the aim of reducing the bias of the results and increasing the reliability and validity of the selected papers. After the search, a total of 89 studies were identified in the first stage. Subsequently, after reading both the title and the abstract by the researchers, 51 studies were eliminated because they did not analyse Boccia as a Paralympic sport. Finally, a total of 38 studies were obtained, published with dates prior to November 2024, the date on which the search was carried out. Figure 1 describes the search process.

### 2.3 Data extraction

For the selection and extraction of information, the Web of Science (WoS) database was used, since it allows obtaining information related to the title, name of the publication (journal), year, abstract, and/or keywords. For this reason, it is one of the most widely used databases for the development of these works (24–26). Specifically, those documents indexed in the WoS Core Collection database were considered, a series of documents only indexed in this electronic platform. VOSviewer plays a pivotal role in visualizing collaboration patterns among countries and authors, facilitating the depiction of co-occurrence networks pertaining to keywords (27).

### 2.4 Data analysis

The laws of bibliometric studies (28) were considered in the development and extraction of data. In addition, different analyses were used for the extraction and interpretation of the results. To evaluate the exponential growth of the number of publications, Price's Law has been used, through the R^2^ coefficient (29), this analysis allows the identification of those obsolete and contemporary documents, closer to the present time. To identify those authors with the greatest number of documents, Lotka's Law (30) was used, through the use of the H index (31). Finally, for the analysis of the key terms used, Zipf's Law (32, 33), was used, including a total of 85 keywords.

Finally, Microsoft Excel (version 2006: Microsoft Corporation, Redmond, WA, USA) and VOSviewer (Center for Science and Technology Studies, The Netherlands) were used for data analysis and visualization. For the creation and visualization of the results, a fragmentation analysis (attraction: 3 and repulsion: −3) was used, according to the topic and temporality of the results (34).

## 3 Results

### 3.1 Analysis of the documents and quotes

Considering the totality of the identified documents, we observed the existence of a study published in 2012, in which the main objective was to analyse the patterns of acute fatigue in neuromuscular activity after a simulated Boccia game, and its effect on sports performance (35). Until 2016, very few manuscripts were published. In detail, no manuscripts were published in 2015, only 1 in 2012, 2013, and 2016, with 2014 being the year with the highest number of publications between 2012 and 2016 (a total of 3). Establishing the study range from 2016 to 2024, an exponential growth of 81.87% of the sample is observed compared to 2012–2016. Notably, 2020 recorded the highest number of publications (*n* = 7). Taking as a reference the number of citations received by year, 2014 has been identified as the year with the highest number of citations (*n* = 42), and 2017 to 2020 the time frame associated with an exponential growth.

**Figure 2:**
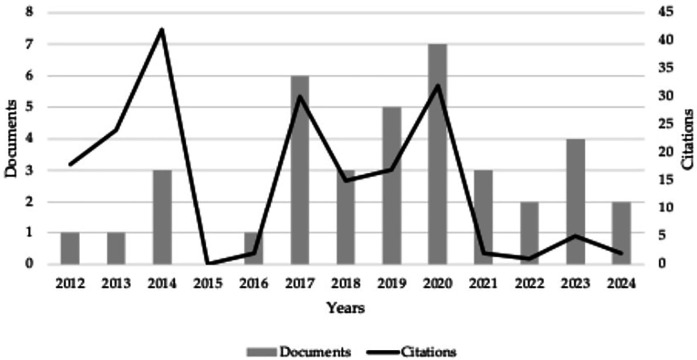
Evolution of documents related to Boccia.

Regarding the reference to the number of citations received in each of the documents, and, after identifying the H index of the selected sample (9), it was observed the existence of two documents with 20 or more citations, more specifically, with a total of 24 (36) and with 23 citations (37). Figure 2 shows the evolution of the documents in the range of dates mentioned above.

Table 1 shows the first 8 documents with the highest number of citations, as well as the average number of citations received per year since the publication. In addition, the resource (journal) in which it was published is shown, as well as the journal's Impact Factor and the Journal Citation Reports (JCR) quartile. This analysis provides the opportunity to identify those journals or platforms with the highest impact index in the scientific community, and to select them to carry out the publications, to obtain greater repercussion of research.

**Table 1:** Most cited documents information.

Authors	Article title	Study's aim	Times cited	Citations per year	Source title	IF	Quartil
de la Vega et al. (36)	Precompetitive Mood and Perceived Performance in Paralympic Boccia	This study examines the mood profile of the Spanish National Boccia team at the Paralympics in Beijing “08. Describes athletic performance perceived by the team, both in individual and collective competitions.	24	2.18	*Revista de Psicologia del Deporte*	0.7	Q4
Huang et al. (37)	Motion Analysis of Throwing Boccia Balls in Children with Cerebral Palsy	This study investigated the differences between children with cerebral palsy and normally developed children regarding throwing patterns of Boccia balls.	23	2.30	*Research In Developmental Disabilities*	2.9	Q1
Fong et al. (35)	Upper Limb Muscle Fatigue during Prolonged Boccia Games with Underarm Throwing Technique	This study investigated the acute fatigue pattern in neuromuscular activity after a simulated Boccia game and the effect of fatigue pattern on sport performance	18	1.50	*Sports Biomechanics*	2.0	Q3
Reina et al. (38)	Throwing Distance Constraints Regarding Kinematics and Accuracy in High-Level Boccia Players	The aim of this study was to investigate whether different throwing distances might have an impact on kinematics and accuracy across repeated throws.	14	2.33	*Science & Sports*	0.8	Q4
Ovenden et al. (16)	Here Everyone is the Same—A Qualitative Evaluation of Participating in a Boccia (Indoor Bowling) Group: Innovative Practice	This qualitative study explored the impact of a Boccia (modified indoor bowls) group on the lives of people with dementia and their careers.	12	2.40	*Dementia International Journal of Social Research and Practice*	2.4	Q2
Tsai et al. (39)	Seat Surface Inclination may affect Postural Stability during Boccia Ball Throwing in Children with Cerebral Palsy	The aim of the study was to examine how seat surface inclination affects Boccia ball throwing movement and postural stability among children with cerebral palsy (CP).	12	1.20	*Research In Developmental Disabilities*	2.9	Q1
Roldan et al. (40)	Inter-Rater Reliability, Concurrent Validity and Sensitivity of Current Methods to Assess Trunk Function in Boccia Players with Cerebral Palsy	Trunk function is a core factor to allocate Boccia players with cerebral palsy in BC1 and BC2 sport classes, according to the Boccia International Sports Federation (BISFed). However, the appropriateness of the current test to assess trunk function has never been studied to determine its reliability, validity and sensitivity to discriminate between different levels of impairment.	12	3.00	*Brain Sciences*	2.7	Q3
Houlihan and Chapman (41)	Talent Identification and Development in Elite Youth Disability Sport	The paper examines the talent identification and development process in three youth disability sports: wheelchair basketball, Boccia and disability tennis. The analysis is concerned to explore the extent of convergence in processes between disability sports and between disability and mainstream sports.	12	1.71	*Sport In Society*	1.5	Q3

### 3.2 Type of documents

From the total number of studies (*n* = 38), a large spectrum of documents can be observed, which have been classified according to their structure and object of realization. For this purpose, the classification established by Gamonales et al. (42) has been established as a reference. This fact allows us to perform a classification of the studies according to their purpose and structure, with “Article” being the most used format (*n* = 22), followed by “Congress” (*n* = 8), whose purpose is to perform the presentation of papers in congresses for greater scientific dissemination. Table 2 shows the number of documents for each item. The total number is higher with the inclusion in the review because certain studies can be placed in different categories.

**Table 2:** Classification of documents.

Type of document	Documents	%
Article	22	68.75
Congress	8	25.00
Book chapter	2	6.25
Early access	1	3.12
Meeting abstract	1	3.12
Review article	1	3.12

### 3.3 Network between authors of documents

**Figure 3:**
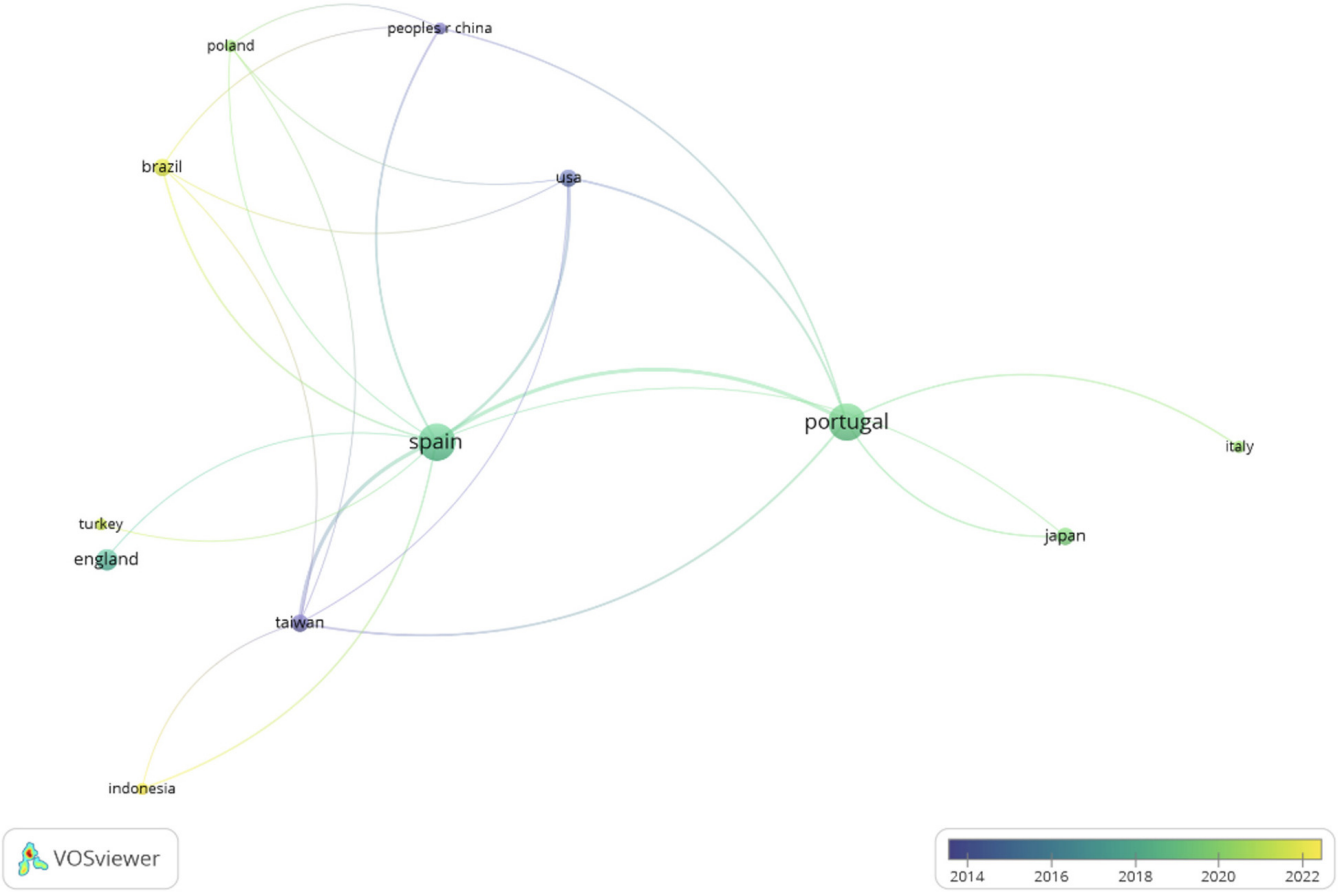
Co-citation of the authors.

Concerning the different authors who have contributed to the development of the different works, a total of 119 authors were associated with 38 selected documents, with an average of 3.6 authors per document, which suggested international collaboration. After a thorough analysis of the authors, it was observed that only 3 authors (Novais, P.; Silva, V.; Soares, F.) have carried out a minimum of 4 studies related to Boccia. On the contrary, considering the number of citations as the main variable of analysis, it is Raúl Reina and Alba Roldán who present the highest number (*n* = 28), with 3 published studies. Figure 3 shows the correlations between the authors, as well as the different groups identified.

### 3.4 Publications considering the country and language

Of all the countries included in the analysis (*n* = 15), Portugal and Spain are in the first position with the highest number of documents (*n* = 9). Table 3 shows the number of documents and citations received for each of the selected countries, and it can be observed that Spain, despite having the same number of published documents, has more than twice the number of citations compared to Portugal (58 vs. 17).

**Table 3:** Classification of the documents considering the country of the first author.

Countries	Documents	%	Citations
Portugal	9	28.12	17
Spain	9	28.12	58
England	3	9.37	11
Brazil	2	6.25	2
Japan	2	6.25	8

After performing a linguistic analysis of the different studies, it was found that 90.6% of the studies were written in English and 9.4% in Spanish.

**Figure 4:**
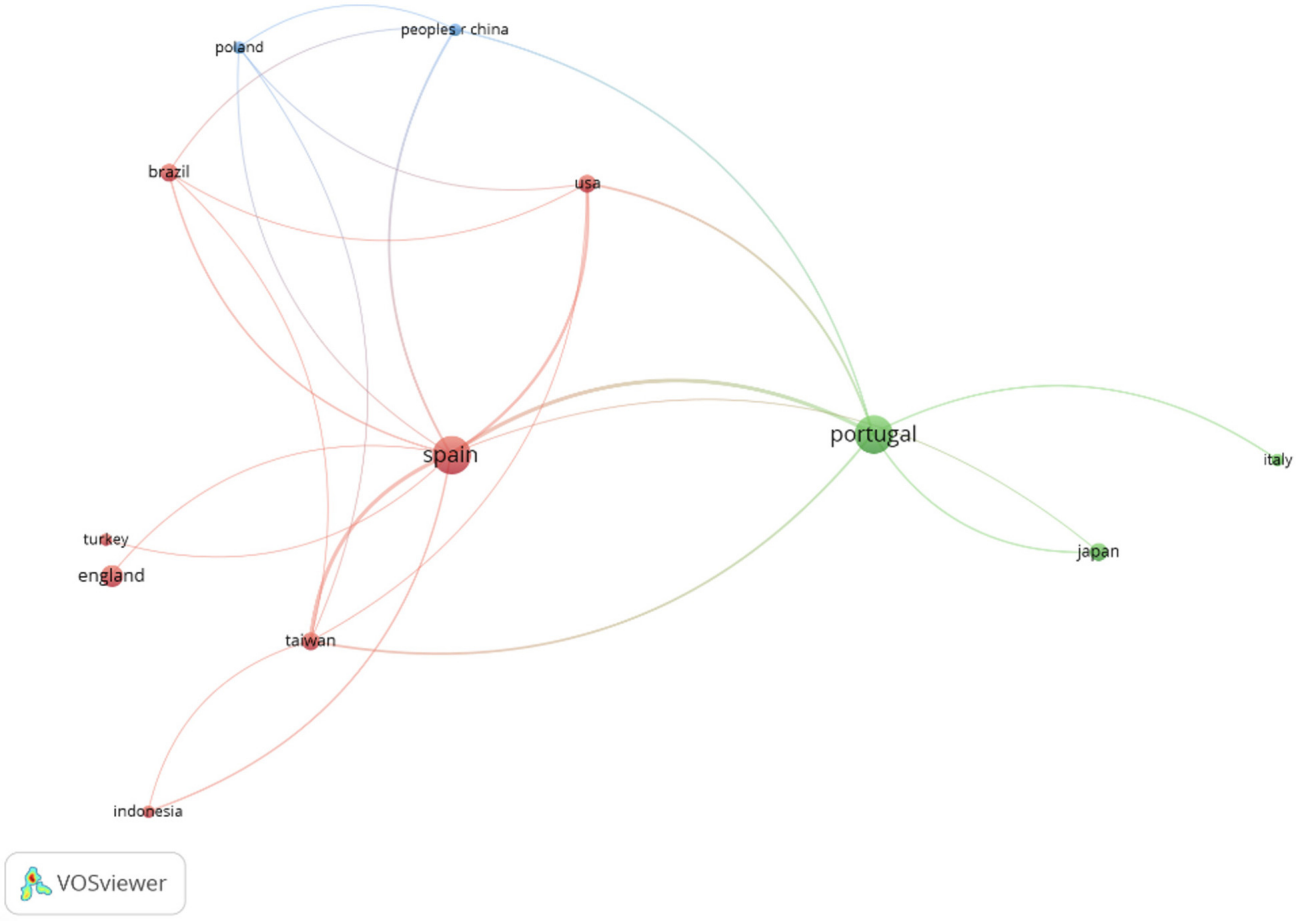
Network of countries.

**Figure 5:**
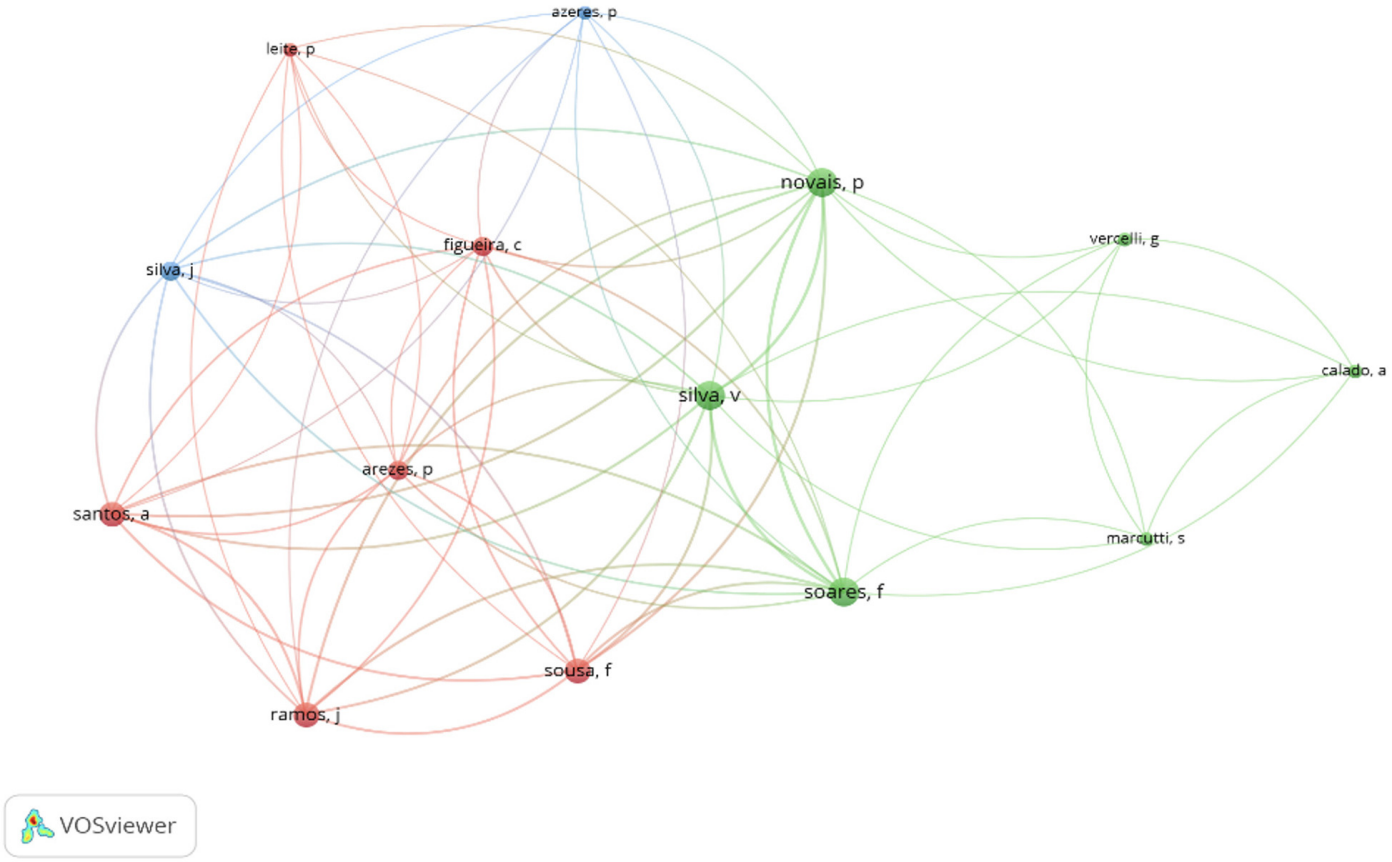
Network of countries considering the temporality.

Figure 4 shows the relationships between the different countries and Figure 5 highlights that Brazil and Indonesia are the countries with the most recent scientific production.

### 3.5 Analysis of the publishers and journals

**Figure 6:**
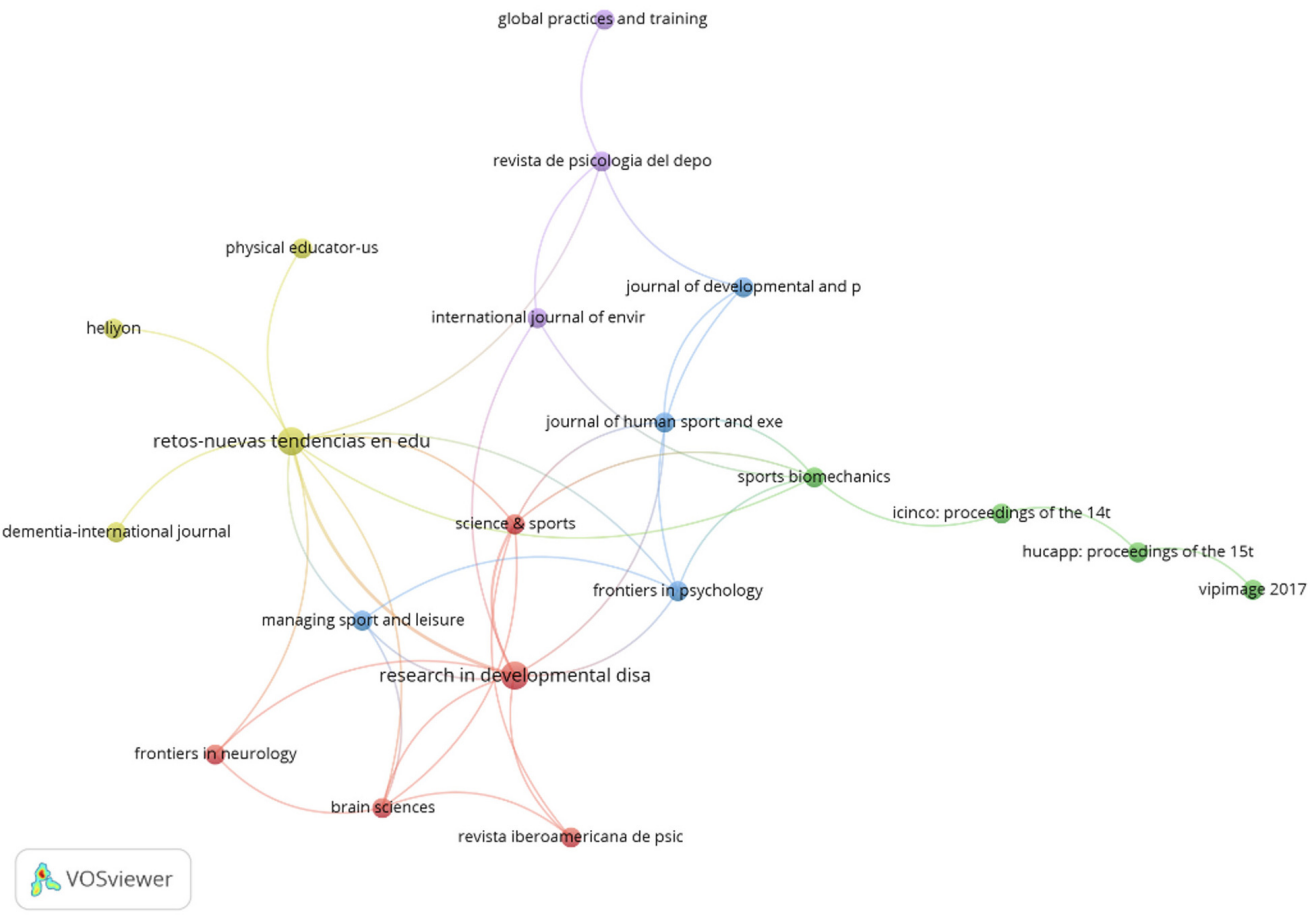
Interactions between journals according to the citations.

About the journals that published the studies, there was a great disparity in the results, since a total of 29 resources were identified, highlighting a wide range of possibilities to publish research related to Boccia. *Springer Nature* was the publisher with the highest number of studies indexed in its platforms (*n* = 7), followed by *Elsevier* (*n* = 4) (Table 4). Similarly, considering the number of citations, *Research in Developmental Disabilities* was identified as the journal with the highest number of citations (*n* = 27), followed by *Revista de Psicología del Deporte* (*n* = 17). This information allows us to identify the platforms with more development of studies related to Boccia, as well as those works with the greatest impact. Figure 6 depicts the interactions between each journal according to the citations.

**Table 4:** Publishers and journals associated with the published documents.

Publishers	Documents	%	Journal	Documents
*Springer Nature*	7	21.87	*Recent Advances in Information Systems and Technologies*	2
*Elsevier*	4	12.50	*Research in Developmental Disabilities*	2
*Taylor & Francis*	3	9.37	*Managing Sport and Leisure*	1
*Federacion Espanola Asociación Docentes Educacion Fisica-Feadef*	2	6.25	*Retos-Nuevas Tendencias en Educacion Fisica Deporte Y Recreacion*	2
*Frontiers Media Sa*	2	6.25	*Frontiers in Psychology*	1

### 3.6 Keywords used by authors

**Figure 7:**
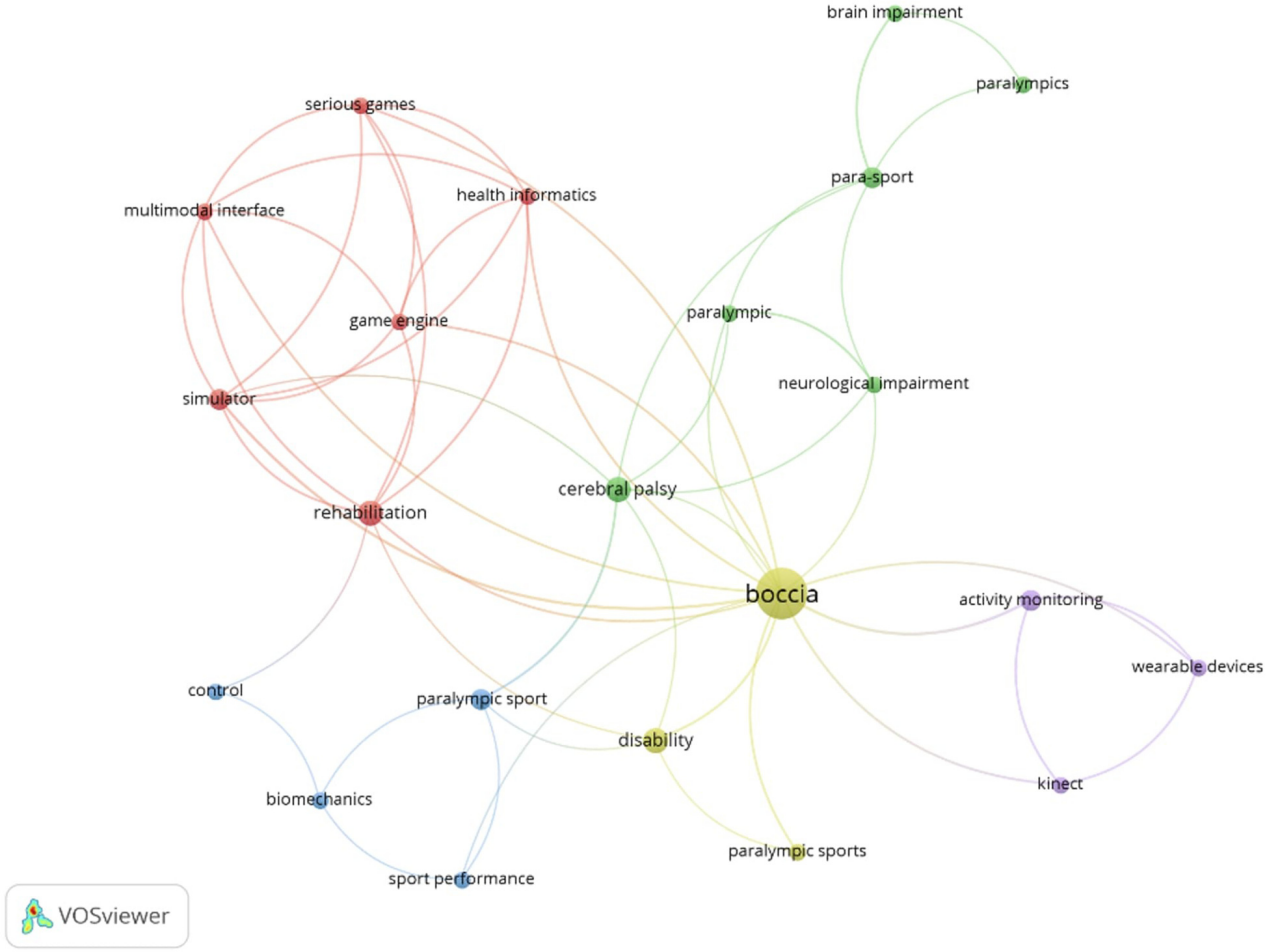
List of keywords used by the authors.

A total of 92 keywords were identified. The terms with the highest occurrence were “boccia” (*n* = 17), “rehabilitation” (*n* = 4), and “disability” (*n* = 4) (Figure 7).

**Figure 8:**
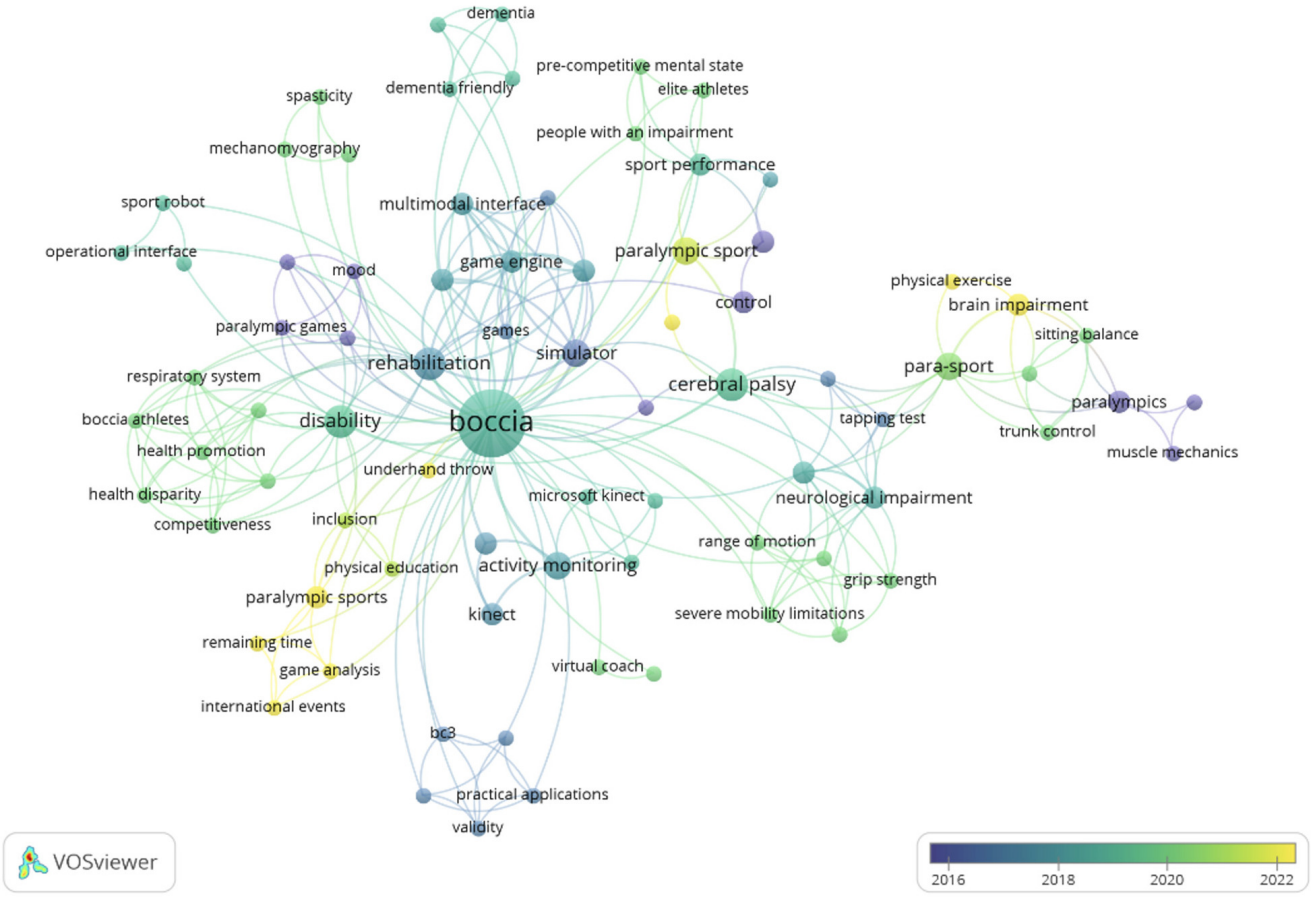
List of keywords based on the year of publication.

According to the temporality of the terms, there is a clear change in the keywords selected by the authors, focusing on the study of specific elements such as “physical exercise”, “Paralympic sport”, “game analysis” or “underhand throw”, with the aim of understanding and improving the performance of Boccia players (Figure 8).

## 4 Discussion

The purpose of this study was to develop a bibliometric review of literature related to Boccia, aiming to analyse relevant information related to the adapted sport, and determine the existing relationships/networks between the different authors and countries, as well as methodological procedures related to publications. Between 2012 and 2016, the highest number of publications per year was 3, in 2015. From 2017 to 2024, an exponential growth of 81.87% of the sample was observed comparing to 2012–2016, with particular emphasis from 2017 to 2020 (in this last year 7 publications), fact that we relate to the COVID-19 pandemic, which limited the involvement and participation in adapted sports, and consequently the access to data for research, observed in our results by the decreasing number of citations between 2020 and 2022. The COVID-19 pandemic period negatively impacted adapted sports even on a greater scale compared to sports because in adapted sports, such as Boccia, participants are more vulnerable, as previously stated by Durstine et al. (43).

Aiming for good sport training for people with disabilities, programmes should focus on flexibility, balance, accessibility, safety, enjoyment, cardiovascular endurance, agility, and muscular strength. Considering the number of citations received by year, 2014 has been identified as the moment with the highest number (*n* = 42). Still, between 2017 and 2020, a high and constant level of citations was observed, evidence that we attribute to the relationship between number of publications and citations (e.g., in 2014, 2017, 2020 and 2023 it was observed an increase in publications and commitment increase in citations), which we relate to self-citations, since they play an important role in the increase of the visibility (44). Articles and congresses were the two most found forms of dissemination, underlining the desire to find and share knowledge in Boccia, despite being in small numbers. This parasport is increasingly becoming recognized as a legitimate component of international sport, although we agree with DePauw and Gavron (45), who previously stated that in some instances the elite-level athletes with disabilities are still not taken as seriously as elite-level able-bodied athletes, and this has consequences in scientific knowledge and dissemination.

We observed a total of 119 authors associated with the 38 selected documents, an average of 3.6 authors per document, but only 3 authors (Novais, P.; Silva, V.; Soares, F.) associated with 4 studies related to Boccia. Regarding citations, Raúl Reina and Alba Roldán are the authors who present the highest number (*n* = 28) with 3 published studies. This evidence relates to another interesting finding, of all the countries included in the analysis (*n* = 15), Portugal and Spain are in the first position with the highest number of documents (*n* = 9), but Spain has more than the double of citation compared to Portugal (58 vs. 17). In our perspective, the level of Boccia in these two countries is very high considering the participation and results in international events and that led to the need of scientific support to practice, hence, the status of the countries in Boccia drove researchers to follow their scientific work in the adapted sport. Other countries with tradition and recent results in this parasport are Brazil and Indonesia, which we found to be the countries with the most recent scientific production. Nevertheless, 90.6% of the studies were written in English and 9.4% in Spanish, which is a challenge for the future because in some of the countries with more tradition in Boccia people speak and read preferably in Portuguese and Spanish.

It was previously stated that it is important to select and publish in journals with a high impact factor and use provocative titles (46). In this study, we found a total of 29 resources, particularly *Springer Nature* was the publisher with the highest number of studies indexed in its platforms (*n* = 7), followed by *Elsevier* (*n* = 4). Considering the number of citations, *Research in Developmental Disabilities* was identified as the journal with the highest number of citations (*n* = 27), followed by *Revista de Psicología del Deporte* (*n* = 17). Boccia is a growing movement parasport, practiced by more and more people, nevertheless, scientific support is still scarce, the number of journals and special issues related to adapted sport is much smaller compared for example to Sport and Exercise and Health, and this represents a challenge to dissemination.

Finally, we identified a total of 92 keywords, the terms with the highest occurrence were “boccia” (*n* = 17), “rehabilitation” (*n* = 4), and “disability” (*n* = 4). According to the temporality of the terms, there was a clear change in the keywords selected by the authors, focusing on the study of specific elements such as “physical exercise”, “Paralympic sport”, “game analysis” or “underhand throw”. Benito-Peinado et al. (47), indicated that in order to increase the visibility and impact of the study in future searches, it is important to select the proper keywords, from the results in this study, Boccia is still linked to the area of rehabilitation rather than performance, one of the challenges for the future, since sports performance has developed in practitioners but not received adequate scientific support.

One of the limitations of the study is the selection of key terms. For the correct development of the study, as well as in the extraction of the results, it was necessary to select those words that are closest to the topic and, in this way, eliminate biases in the results, therefore, ensuring that the studies identified were specifically related to the study topic. On the other hand, one of the strengths of the study is that it allows us to understand the trends and co-authorships of the documents regarding the country or authors. For future research, it is proposed to analyse other areas closely related to Boccia, considering other keywords and databases along with the WoS. The analysis of the technical and tactical details associated with performance should shed light on the game dynamics and improve training and competitive strategies.

## 5 Conclusions

Despite scientific evidence that Boccia is important for the individual global well-being, and inclusive of all genders, ages, and abilities, the findings revealed that the initial document related to Boccia and focusing the Paralympic status was published in 2012, after being introduced as Paralympic sport in 1984. This study confirms the scarcity of research in Boccia, highlighting that Portugal and Spain have been the most prolific countries over the years, with their researchers also associated with more citations.

From our perspective, more journals and special issues in sports journals related to the study topic are necessary to provide the opportunity to support the practice with scientific knowledge, something that our results found between 2017 and 2020, but was afterward changed (in our perspective due to the pandemic phase). At this stage, more and better research is needed, from our point of view in other languages different than English to provide the opportunity not only to higher access by non-English native, but also in the perspective of establishing networks, which seems to be starting to materialize when we look at the countries most focused on research on this subject at the moment (Brazil and Indonesia).

Deepening knowledge in this study topic will allow an increasing number of countries and authors to be involved, and in this way improve the practice, training, and competitive performance in this adapted sport, along with the improvement in the quality of life of the population.

## 6 Practical applications

In essence, this research is crucial for the identification of trends and networks related to the study of Boccia in the context of Paralympic sport. Demonstrates the need for more applicable research in different perspectives of the game by the scientific community, returning to the growing interest before the pandemic. The findings of this study provide guidance for future investigations, sport rules, social and sport policies, and the creation of focused interventions aimed at encouraging a healthier and more inclusive society.

## Data availability statement

The data that supports the findings of this study are available from the first and last authors, upon reasonable request.

## References

[1] Blauwet C Willick SE. The paralympic movement: using sports to promote health, disability rights, and social integration for athletes with disabilities. PM R. (2012) 4(11):851–6. 10.1016/j.pmrj.2012.08.015 23174549

[2] Phytanza DTP Burhaein E Lourenço CCV Pavlovic R. Physical activity based on manipulative exercise: how it affects the gross motor of children with autism for 12 years old? Int J Disabil Sports Health Sci. (2023) 6(2):171–80. 10.33438/ijdshs.1258177

[3] Yazicioglu K Yavuz F Goktepe AS Tan AK. Influence of adapted sports on quality of life and life satisfaction in sport participants and non-sport participants with physical disabilities. Disabil Health J. (2012) 5(4):249–53. 10.1016/j.dhjo.2012.05.003 23021735

[4] Demirci N Pinru Phytanza DT. Investigation of obesity, physical activity and sedentary behaviors of individuals with and without autism spectrum disorder during the COVID-19 pandemic process. JUMORA: Jurnal Moderasi Olahraga. (2021) 1(02):45–55. 10.53863/mor.v1i02.220

[5] Phytanza DTP Burhaein E Lourenço CCV Andika A. The effect of net play on forearm passing ability on junior high school level inclusion schools. J Hum Move Sports Sci. (2022) 10(5):1067–74. 10.13189/saj.2022.100525.

[6] Burhaeın E Phytanza DTP Lourenço CCV Abror M Setiawan A. Adapted physical education for autism spectrum disorder: a bibliography analysis in publication 2001–2023. Int J Disabil Sports Health Sci. (2023) 6(3):364–72. 10.33438/ijdshs.1300114

[7] Coutinho RX Acosta MA. Male environments of third age. Cien Saude Colet. (2009) 14(4):1111–8. 10.1590/S1413-81232009000400017 19721951

[8] Atasoy T Pekel A. The relationship between quality of life level and social appearance anxiety level of physically handicapped boccia athletes. Phys Educ. (2021) 78(1):1–10. 10.18666/TPE-2021-V78-I1-10352.

[9] Barak S Mendoza-Laiz N Fuentes MTG Rubiera M Huyzler Y. Psychosocial effects of competitive Boccia program in persons with severe chronic disability. J Rehabil Res Dev. (2016) 53(6):973–88. 10.1682/JRRD.2015.08.0156 28475199

[10] Ferreira C Gamonales JM Aleluia M Oliveira A Pardana F Santos F Boccia in paralympic games: the evolution from 1984 to 2016 and future perspectives. Cuadernos de Psicologia Del Deporte. (2022) 22(1):205–14. 10.6018/cpd.492031

[11] Ferreira CC Gamonales JM Espada MC Muñoz-Jiménez J. The influence of Boccia classification on the performance in international-level events. E-Balonmano Com. (2025) 21(2):277–90. 10.17398/1885-7019.21.277

[12] BISFed. About Boccia. BISFed Classification, Boccia Classification Rules. London: Boccia International Sport Federation (2021). Available at: https://www.worldboccia.com/wp-content/uploads/2021/10/Boccia-Classification-Rules-5th-Edition-September-2021287.pdf (Accessed February 25, 2025).

[13] Ferreira CC Gamonales JM Muñoz-Jiménez J Espada MC. Gender participation and performance in boccia international-level events. J Funct Morphol Kinesio. (2025) 10(1):87. 10.3390/jfmk10010087 PMC11943150 40137339

[14] Siavoshy H Bolurian F. The effects of 12 weeks of playing boccia on the social development of children with cerebral palsy and intellectual disability. J Except Child. (2016) 15(4):45–51.

[15] Sobiecka J Plinta R Kądziołka M Gawroński W Kruszelnicki P Zwierzchowska A. Polish Paralympic sports in the opinion of athletes and coaches in retrospective studies. Int J Environ Res Public Health. (2019) 16(24):4927. 10.3390/ijerph16244927 31817445 PMC6950017

[16] Ovenden I Dening T Beer C. “Here everyone is the same”—a qualitative evaluation of participating in a Boccia (indoor bowling) group: innovative practice. Dementia. (2019) 18(2):785–92. 10.1177/1471301216675988 27771615

[17] Calado A Marcutti S Silva V Vercelli G Novais P Soares F. Towards a virtual coach for Boccia: developing a virtual augmented interaction based on a Boccia simulator. In: Proceedings of the 15th International Joint Conference on Computer Vision, Imaging and Computer Graphics Theory and Applications—volume 2: HUCAPP, ISBN 978-989-758-402-2 (2020). p. 217–24. 10.5220/0009142602170224

[18] Williamson JA McDonald FW Galligan EA Baker PG. Selection and training of disabled persons for scuba-diving: medical and psychological aspects. Med J Aust. (1984) 141:414–8. 10.5694/j.1326-5377.1984.tb132849.x 6236353

[19] Ferreira C Gamonales J Espada M Muñoz-Jiménez J. Current status of sport performance in Boccia: systematic review of the literature. Retos. (2023) 48:1070–7. 10.47197/retos.v48.95110

[20] Montero I León OG. A guide for naming research studies in psychology. Int J Clin Health Psychol. (2007) 7(3):847–62.

[21] Ato M López-García JJ Benavente A. A classification system for research designs in psychology. Ann Psychol. (2013) 29(3):1038–59. 10.6018/analesps.29.3.178511.

[22] van Eck NJ Waltman L. Citation-based clustering of publications using CitNetExplorer and VOSviewer. Scientometrics. (2017) 111(2):1053–70. 10.1007/s11192-017-2300-7 28490825 PMC5400793

[23] Maier D Maier A Așchilean I Anastasiu L Gavriș O. The relationship between innovation and sustainability: a bibliometric review of the literature. Sustainability. (2020) 12(10):4083. 10.3390/su12104083

[24] Cabo CA Hernández-Beltrán V Gamonales JM Fernandes O Espada MC Parraca JA. Evolution of documents related to the influence of physical activity and functional capacity throughout the aging process: a bibliometric review. Front Physiol. (2024) 15:1427038. 10.3389/fphys.2024.1427038 39156828 PMC11327041

[25] Cabo CA Hernández-Beltrán V Gamonales JM Parraca JA Fernandes O Espada MC. Evolution of research related to how a sedentary lifestyle influences the aging process: a bibliometric review. J Public Health. (2024) 10.1007/s10389-024-02327-7 PMC11327041 39156828

[26] Hernández-Beltrán V Espada MC Castelli Correia de Campos LF Gamonales JM Becerra-Patiño BA. Analysis of the publications related to sports performance in wheelchair basketball: bibliometric review. Retos. (2024) 61:344–55. 10.47197/retos.v61.105959

[27] Yan L Chen Z Zhang X Han Q Zhu J Wang Q Themes and trends in marathon performance research: a comprehensive bibliometric analysis from 2009 to 2023. Front Physiol. (2024) 15:1388565. 10.3389/fphys.2024.1388565 38798878 PMC11116898

[28] Contreras-Barraza N Madrid-Casaca H Salazar-Sepúlveda G Garcia-Gordillo MÁ Adsuar JC Vega-Muñoz A. Bibliometric analysis of studies on coffee/caffeine and sport. Nutrients. (2021) 13(9):3234. 10.3390/nu13093234 34579111 PMC8466917

[29] Price DDS. A general theory of bibliometric and other cumulative advantage processes. J Am Soc Inf Sci. (1976) 27(5):292–306. 10.1002/asi.4630270505

[30] Coile RC. Lotka’s frequency distribution of scientific productivity. J Am Soc Inf Sci. (1977) 28(6):366–70. 10.1002/asi.4630280610

[31] Hirsch JE. An index to quantify an individual’s scientific research output. Proc Natl Acad Sci USA. (2005) 102(46):16569–72. 10.1073/pnas.0507655102 16275915 PMC1283832

[32] Valderrama-Zurián JC García-Zorita C Marugán-Lázaro S Sanz-Casado E. Comparison of MeSH terms and KeyWords plus terms for more accurate classification in medical research fields. A case study in cannabis research. Inf Process Manag. (2021) 58(5):102658. 10.1016/j.ipm.2021.102658

[33] Vega-Muñoz A Salazar-Sepúlveda G Contreras-Barraza N Araya-Silva L. Scientific mapping of coastal governance: global benchmarks and trends. J Mar Sci Eng. (2022) 10(6):751. 10.3390/jmse10060751

[34] Uribe-Toril J Ruiz-Real J Haba-Osca J de Pablo Valenciano J. Forests’ first decade: a bibliometric analysis overview. Forests. (2019) 10(1):72. 10.3390/f10010072

[35] Fong DT-P Yam K-Y Chu VW-S Cheung RT-H Chan K-M. Upper limb muscle fatigue during prolonged Boccia games with underarm throwing technique. Sports Biomech. (2012) 11(4):441–51. 10.1080/14763141.2012.699977 23259234

[36] de la Vega R Galan A Ruiz R Tejero C. Precompetitive mood and perceived performance in Paralympic Boccia. Rev Psicol Deporte. (2013) 22(1):39–45.

[37] Huang P-C Pan P-J Ou Y-C Yu Y-C Tsai Y-S. Motion analysis of throwing Boccia balls in children with cerebral palsy. Res Dev Disabil. (2014) 35(2):393–9. 10.1016/j.ridd.2013.11.017 24334228

[38] Reina R Domínguez-Díez M Urbán T Roldán A. Throwing distance constraints regarding kinematics and accuracy in high-level boccia players. Sci Sports. (2018) 33(5):299–306. 10.1016/j.scispo.2018.03.078

[39] Tsai Y-S Yu Y-C Huang P-C Cheng H-YK. Seat surface inclination may affect postural stability during Boccia ball throwing in children with cerebral palsy. Res Dev Disabil. (2014) 35(12):3568–73. 10.1016/j.ridd.2014.08.033 25241116

[40] Roldan A Barbado D Vera-Garcia FJ Sarabia JM Reina R. Inter-rater reliability, concurrent validity and sensitivity of current methods to assess trunk function in boccia players with cerebral palsy. Brain Sci. (2020) 10(3):130. 10.3390/brainsci10030130 32110853 PMC7139471

[41] Houlihan B Chapman P. Talent identification and development in elite youth disability sport. Sport Soc. (2017) 20(1):107–25. 10.1080/17430437.2015.1124566

[42] Gamonales JM Muñoz-Jiménez J León K Ibáñez SJ. 5-a-side Football for individuals with visual impairments: a review of the literature. Eur J Adapt Phys Act. (2018) 11(1):4. 10.5507/euj.2018.004

[43] Durstine JL Painter P Franklin BA Morgan D Pitetti KH Roberts SO. Physical activity for the chronically ill and disabled. Sports Med. (2000) 30(3):207–19. 10.2165/00007256-200030030-00005 10999424

[44] González-Sala F Osca-Lluch J Haba-Osca J. Are journal and author self-citations a visibility strategy? Scientometrics. (2019) 119(3):1345–64. 10.1007/s11192-019-03101-3

[45] DePauw K Gavron S. Disability Sport. 2nd ed. Champaign, IL: Human Kinetics Publishers (2005). 10.5040/9781492596226

[46] Araujo AC Nascimento DP Gonzalez GZ Costa LOP. How to increase the visibility of scientific articles through social media? Braz J Phys Ther. (2018) 22(6):435–6. 10.1016/j.bjpt.2018.08.009 30146107 PMC6235750

[47] Benito-Peinado P Díaz-Molina V Calderón-Montero F Peinado-Lozano A Martín-Caro C Árlvarez-Sánchez M Literature review in exercise physiology: practical re-commendations. Revista Científica Internacional de Ciencias de la Actividad Física y del Deporte. (2007) 6(3):1–11. 10.5232/ricyde2007.00601.

